# Outcomes of hospitalized hematologic oncology patients receiving rapid response system activation for acute deterioration: another time, another way

**DOI:** 10.1186/s13054-019-2714-0

**Published:** 2019-12-23

**Authors:** Belén Civantos, José Manuel Añón, Santiago Yus, María José Asensio, Abelardo García-de-Lorenzo

**Affiliations:** 1 Servicio de Medicina Intensiva, Hospital Universitario La Paz-Carlos III-Cantoblanco, IdiPAZ, Paseo de la Castellana 261, 28046 Madrid, Spain; 2 CIBER de Enfermedades Respiratorias, Instituto de Salud Carlos III, Madrid, Spain

**Keywords:** Malignancy, Critical care, Hematology, Mortality

Dear Editor,

We have read the recent article written by Gershkovich et al. [1]. In this paper, the authors sought to examine the outcomes of hospitalized hematologic oncology patients requiring rapid response system (RRS) activation after clinical deterioration and to identify the factors that are independently associated with in-hospital mortality. The authors concluded that hematologic oncology patients who are admitted to the hospital and suffer an acute deterioration experience high rates of ICU admission and in-hospital mortality.

In recent years, there has been an increase in the number of hematologic oncology patients admitted to ICUs. Currently, this condition is no longer a criterion to dismiss admission to the ICU [2]. Due to the continuous increase in the number of these patients and the complexity of their disease, we developed in 2012 a new strategy to optimize the management of this type of patients. We considered that RRS did not reflect accurately the severity of their condition. To date, the role of these alert systems is not yet clearly established [1].

Our strategy is based on daily multidisciplinary ward rounds with hematologists and intensivists in order to identify the high-risk patients and to admit them early to the ICU. The association between early versus late ICU admission and improved survival in hematologic oncology patients has been previously established [3]. Our data before (2000–2011) and after (2012–2016) implementation shows the following:

1. An 8% increase in the total number of admissions to critical care after implementation
2. Reduction of in-ICU mortality, 90-day mortality, and post 90-day mortality (44%, 54%, and 62% in the 2012–2016 period) as compared with the pre-implementation period (53%, 61%, and 66%)

According to Azoulay et al. [3], teamwork and high-quality communication between hematologists and intensivists improve patient management. In our center, this new strategy of early admission of high-risk hematologic oncology patients resulted in a higher number of admissions and a lower mortality both in the ICU and after ICU and hospital discharge.

These promising outcomes have led to a new collaboration agreement with the Oncology Department with the final aim of replicating these results in patients with solid tumors.

Since the RRS has not yet demonstrated improved outcomes in the hematologic oncology patients, we believe that our model could be a valid alternative to those by facilitating an early admission to critical care, improving outcomes and being cost neutral.

## Data Availability

The datasets during and/or analyzed during the current study are available from the corresponding author on reasonable request.

## References

[1] Gershkovich B, Fernando SM, Herritt B, Castellucci LA, Rochwerg B, Munshi L (2019). Outcomes of hospitalized hematologic oncology patients receiving rapid response system activation for acute deterioration. Crit Care.

[2] Azoulay E, Soares M, Darmon M, Benoit D, Pastores S, Afessa B (2011). Intensive care of the cancer patient: recent achievements and remaining challenges. Ann Intensive Care.

[3] Azoulay E, Pène F, Darmon M, Lengliné E, Benoit D, Soares M (2015). Managing critically ill hematology patients: time to think differently. Blood Rev.

